# Acceptability of Health Information Technology by Health Care Professionals: Where We Are Now and How We Can Fill the Gap

**DOI:** 10.2196/72184

**Published:** 2025-12-02

**Authors:** Corinne Isnard-Bagnis, Stéphane Mouchabac, Riadh Lebib, Hervé Bismut, Pierre Alexis Geoffroy

**Affiliations:** 1 Nephrology Department, Pitié-Salpêtrière Hospital, AP-HP, Sorbonne Université, 47-83 boulevard de l'Hôpital, Paris, 75013, France, 33 1-4217-7211; 2 Academic Digital Medical Hub, AP-HP, Paris, France; 3 Department of Psychiatry, Saint-Antoine Hospital, AP-HP, Sorbonne Université, Paris, France; 4 Infrastructure for Clinical Research in Neurosciences (iCRIN), Paris Brain Institute, Paris, France; 5 Humans Matter, Paris, France; 6 Geminicis, Paris, France; 7 Département de psychiatrie et d'addictologie, AP-HP, GHU Paris Nord, DMU Neurosciences, Hôpital Bichat-Claude-Bernard, Paris, France; 8 Centre ChronoS, GHU Paris psychiatrie & neurosciences, Paris, France; 9 Université Paris Cité, Inserm, NeuroDiderot, Paris, France

**Keywords:** digital health, electronic health report, artificial intelligence, burden, burn-out, acceptability

## Abstract

Digital health is expected to improve the efficiency and quality of health. Health information technologies (HIT) imply allocated time, appropriate training, and new types of responsibility, whose physical and mental impact on health care professionals (HCPs) has emerged as an important issue. The present review provides updated data and opinions about such potential impact and discusses the relevance of programs established to better characterize barriers and facilitators of HIT implementation. The extent of internet-based health care information and digital apps imposes new responsibilities on HCPs in helping patients select reliable sources and incorporate them in the understanding and self-management of the disease. Several reviews also identified exhaustion, depersonalization, workload, over-alerting, poor work-life integration, and job unsatisfaction as potential drivers of electronic health record (EHR)-associated clinician burnout and HIT unacceptability. Paradoxically, the increasing use of generative artificial intelligence (AI) in the decision-making process may in turn introduce an additional layer of complexity due to required specific skills and associated cognitive overload and stress. Regarding EHRs, various approaches like more proportionate use, better adequation of available commercial tools, or multidisciplinary workflows within the clinic and building of new specialty-specific tools are expected to reduce clinician burden. Studies that focused on EHR paved the way for further multidisciplinary projects designed to define the factors and dimensions impacting overall digital environment including AI, and to identify relevant ways of optimizing its acceptability by HCPs. The way of preventing and alleviating the adverse effects of digital health is a major challenge that all HIT stakeholders should be aware of.

## Introduction

Over the past two decades, digitization has rapidly become the norm across most domains of life. In health care, digital integration has taken multiple forms. Mobile apps, often coupled with internet access, have become essential tools in the management of patients with chronic diseases [1-4] and even cancer [5,6]. The recent COVID-19 pandemic highlighted the benefits and feasibility of digital tools to promote patient empowerment and literacy [7,8] and to optimize and share decision-making by using electronic health records (EHRs) [9]. From the perspective of health care professionals (HCPs), health-information technology (HIT) including artificial intelligence (AI) is expected to improve the efficiency and quality of health as a complementary approach [10].

However, while the increasing use of HIT to improve access to information, its storage, and its sharing among HCPs or with patients is promising, such an intervention warrants an evidence-based evaluation of the overall benefits and potential harms and limits [10-13]. The benefits of digital tools applied to health care, and their limits in terms of reliability and potential malpractice liability associated with AI, have been largely discussed in recent papers highlighting the need for guidelines for assessing and implementing available technology in patients’ management [4,14,15]. AI-related ethical issues regarding, for instance, the valorization of HCP skill should also be considered.

Once it is acknowledged that digital interventions will complement traditional patient follow-ups, the time required for training and effectively integrating digital tools into clinical practice becomes a major issue that should not be underestimated [16,17]. Additionally, the development of a specific digital therapeutic alliance—fostering trust and engagement between patients and health care providers in the digital realm—will be crucial to ensuring the success of these interventions [18]. Several studies and reports have recently addressed the impact of digital interventions on HCPs, with a particular focus on the use of EHRs [19-23], and have suggested a need for recommendations and rules. In that respect, the British National Health System identified key pillars to make digital interventions sustainable for HCPs, which include clinical and behavioral insights, process engineering, and knowledge management [13]. Furthermore, some programs have emerged to optimize EHR efficiency and mitigate EHR-associated burdens [21,22,24,25].

Beyond EHRs whose impact on HCPs is well documented, other health-related digital tools that are expected to make patient management more rapid and efficient, including those related to imaging or tele-surveillance [15], deserve to be considered for their possible direct (stress) and indirect (fatigue) mental impacts on physicians. AI-associated medical errors and their impact on HCP mental health are also a major ethical issue. In this context, the way to better characterize the impact of the various HIT-associated tools—to mitigate this impact on the example of emerging initiatives and to improve the acceptability of HIT implementations—remains an important gap to be addressed.

## Challenges for HCPs in the Era of the Digitization of Care

**Figure 1.**
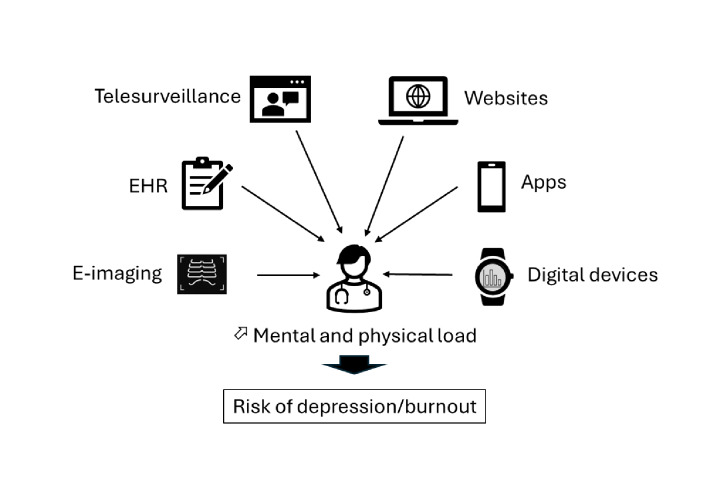
Health digital tools impacting the mental and physical status of health care professionals (HCPs). Digitization in health care includes several types of tools whose benefits in health care are expected to be coupled with an impact on HCPs’ mental and physical status and a risk of burnout. EHR: electronic health record.

The implementation of digitization in health care over the past decades has resulted from several factors related to the rapid development of digital tools and consequently the opportunity to optimize patients’ follow-up and data collection (eg, surrogates of disease evolution and imaging). In return, digital health tools are expected to impact the HCPs’ mental and physical status (Figure 1). The impact of HIT can be addressed in considering three groups: (i) digital tools enabling patients to get and share information with HCPs (eg, via websites or apps); (ii) software like EHRs allowing HCPs to securely store, share, and analyze their patient-related information; and (iii) AI that is expected to help HCPs with decision-making.

## Internet, Mobile Apps, Telemedicine: New Responsibilities for the “Digital Practitioner”

The growth of the internet and the development of digital games have been two major societal hallmarks of the past 30 years. Because patients are part of this changing world, new methods of accessing information and interacting with digital interfaces have inevitably entered the health care domain. The development of health care “gamification” as the result of the widespread use of smartphones and apps has led to concerns about how to appropriately incorporate it in terms of both regulatory processes and the role of HCPs in helping patients use the apps and select effective and safe health care applications [26,27]. Beyond their role as digital practitioners, HCPs should be aware of patients’ behavior towards health care information from the internet and of the methods for informing and educating them on how to access credible, comprehensive, and adapted information [28-31].

Although people use internet-based health care information to different extents [29], data from the Health Information National Trends survey showed that such use generally corresponds to the first place people go to seek information about health or medicine [32]. However, the capacity of the internet to contribute to patients’ knowledge may be limited and sometimes offset by the quality and sources of content. For instance, a recent study assessing 83 websites dedicated to hip and knee arthritis found that most (55%) were of poor quality and that more than half (54%) of them did not mention any risks or complications associated with surgical interventions [31]. Another study assessing websites dedicated to prostate cancer concluded that most were unreliable as lone sources of information for patients [33].

Because most patients diagnosed with a severe disease or followed for a chronic one are expected to get information from the Internet, particularly regarding therapeutic options, HCPs must ensure that patients can differentiate between reliable and unreliable sources. The role of HCPs in this context is to help patients identify credible sources and thus reinforce the patient–HCP relationship. Consequently, HCPs must be aware of the information their patients are likely to encounter and be equipped to offer advice on how to find appropriate and reliable information when patients are researching on their own. Thus, HCPs should focus on several key elements to evaluate the reliability of websites’ medical information: author details (credentials and contact information), scientific background of sources, recency of the information (date of the last review), rationale and relevance of the website or app (versus others on the same topics), possible conflicts of interest, and whether the website is sales oriented [30,34]. These guidelines should be followed by HCPs so they can direct patients to trustworthy websites. Finally, HCPs themselves may be exposed to liability-related issues due to possible telemedicine-based misdiagnoses [14].

Overall, the mental load that is induced by a new type of responsibility coupled with after-hours time is expected to contribute to HCPs’ psychological burden. This applies to the assessment of medical website and app reliability, but also to the management of digital patients’ information.

## EHRs: A Potential Impact on Mental and Physical Load

Another challenge for HCPs is the need to be familiar with digital tools used for patients’ management like imaging software and EHRs. A systematic review of the literature including 31 articles published between 2010 and 2020 identified direct and indirect drivers of EHR-related burnout, which included direct issues like exhaustion and depersonalization as well as efficiency and resources, workload and requested efforts, work-life integration, and organizational values like job satisfaction [35]. Although EHR alerts are expected to improve care processes, over-alerting may also lead to fatigue and subsequent overriding [36]. During the COVID pandemic, the implementation of a systematic approach to the use of EHRs in order to provide a daily summary for clinical practice and an update of individual information was shown to be feasible and useful during this transient period [9]. However, sequential mixed methods based on quantitative (EHR usage patterns) and qualitative (physician perspectives) components highlighted an increase in after-hours EHR usage and documentation time per patient coupled with a decrease in order time per patient from the pre- to the pandemic period [37]. This suggests a need for more flexible EHR systems that minimize after-hours work and promote a better harmony of work and life. Other factors have been identified as contributors of EHR-induced cognitive overload among HCPs, which include non-intuitive visual displays and numerous default settings, navigation-associated unnecessary steps, and risks of errors due to fatigue or managing inbox tasks [23,38]. In that respect, a set of digital health competencies to be embedded in physician training has been proposed, which would be based on digital medical expertise and abilities in terms of judgement/decision-making, communication, quality and safety, cultural and social competence (eg, applying ethical, legal, and regulatory guidelines), teaching and learning (eg, remaining up-to-date in knowledge and skills), research (eg, basic descriptive statistics and evaluation of the role of human-computer collaboration), leadership, management, and teamwork [17].

While there is certainly a significant need to equip HCPs with appropriate competencies, the psychological and emotional issues related to HIT like EHRs, that have increased over the years, should also be taken into consideration [39]. Such integrative approach must be applied to all emerging HIT including AI.

## Generative AI: A Need for a Balanced Integration in Clinical Environments

AI is expected to improve the efficiency and quality of health and care delivery [10]. In psychiatry, for instance, AI appears as an interesting clinical decision-associated tool for diagnosis, decision support, or relapse prediction [40]. Although these tools promise to enhance diagnosis accuracy and patient management, they are also potentially associated with significant psychological and cognitive risks so the increasing use of generative AI in digital-medical solutions introduces an additional layer of complexity for both practitioners and patients. For health care providers, the need to evaluate and oversee AI-generated insights can contribute to cognitive overload, exacerbate stress, and increase the risk of burnout [41,42]. Although generative AI systems, such as ChatGPT, are often praised for their ease of use—requiring minimal prior training and offering immediate results—their long-term impacts on cognitive resources are likely more nuanced.

At first glance, generative AI may seem to reduce mental load by streamlining routine tasks. However, the rapid pace of AI development and the constant need to adapt to new versions can lead to what authors have referred to as “technostress,” which occurs when the continuous evolution of tools outpaces the users’ capacity to keep up [43]. A recent systematic review of large language models (LLMs) in health care underscored this point and revealed that despite such models’ promise, the evaluations of these models in real clinical settings have remained fragmented and insufficient, with only 5% of studies using real patient data [44]. This disconnect between AI’s potential and practical clinical evaluation contributes to a cognitive strain on health care providers, who must navigate these tools in environments where their actual utility has not been fully validated.

Furthermore, the overreliance on AI tools driven by their perceived infallibility threatens critical cognitive faculties such as judgment and decision-making. Research has shown that users often defer to AI-generated outputs without sufficient scrutiny, which may erode critical-thinking skills over time [45]. This risk is particularly pronounced in clinical settings because the need to validate AI-driven decisions can intensify cognitive load and thus undermine the initial perception that these tools reduce mental effort. As highlighted in the systematic review on LLMs based on 519 studies, many studies have narrowly focused on the accuracy of AI models and have neglected critical dimensions such as bias, fairness, and the potential for harmful outputs-dimensions that are vital in safeguarding both practitioners and patients from undue mental burdens [44].

Digital tools and AI in health care are also expected to facilitate the delegation of administrative tasks and ostensibly free up time for health care providers; however, is this additional time directed towards meaningful patient engagement, or does it instead heighten productivity pressures? This ethical consideration raises questions about the distribution of time and the preservation of human connection in prioritizing care. Moreover, the subjective experience of time in therapeutic relationships—encompassing both the provider’s and the patient’s perceptions—may be influenced by AI. Although technology has the potential to enhance personalized care, it may also impose rigid schedules that diminish flexibility.

Furthermore, the paradox of technological acceleration must be addressed. Although AI can create efficiencies, it may also expedite care delivery and thus lead to an increased patient load and potentially compromise the quality of care, which relies on a patient-centered approach. This dynamic further complicates the management of emotional time because the emotional burdens of caregivers remain unmitigated by technology, potentially exacerbating burnout. The collective temporalities within multidisciplinary teams are also affected because AI can streamline information sharing but may disrupt collaborative rhythms. Additionally, the introduction of AI necessitates significant training time for health care professionals and prompts a reassessment of educational programs to prevent adding further burdens. Human oversight in clinical decision-making remains essential; thus, time must be allocated for the careful analysis of AI recommendations to ensure informed decisions. Lastly, because AI usage raises significant ethical issues regarding data privacy and the trust relationship between patients and providers, it is crucial to allocate time for health care teams to engage in discussions about these challenges, thereby ensuring that ethical considerations remain central in this digital transition.

Overall, there is a need for a balanced integration of AI in clinical environments accompanied by ethical guidelines and mental-health support systems to ensure that these technologies serve their intended purposes without compromising the well-being of HCPs.

## Optimization of the Benefits of Health Digitization: Experience Gained Using EHRs

Harnessing the potential of digital tools like EHRs while addressing the methods of overcoming their impacts on HCPs is a major challenge, and several leverages have been proposed by physicians: in-house supports, scribes, organizational adjustment (eg, shared templates and team-based documentation), and HIT-related factors [35]. Incorporating user-friendly interfaces and reducing the complexity of navigation can further alleviate the burden on HCPs. Machine learning to rank patient information and improve EHR workflows, EHR training, and stewardship programs to optimize alert effectiveness have also been addressed [21,23,38,46].

A systematic review of 81 articles identified relevant factors that significantly influenced the effects of the digital environment on HCP burnout and the corresponding leverages [21]. That study highlighted more proportionate use of EHRs (sometimes disproportionate in some specialties), better adequacy of available commercial EHRs, and scribe implementation to decrease physician hours and after-hours. The capacity to reduce EHR burden and increase physician satisfaction was assessed by UCHealth, a large, integrated health network in the University of Colorado [24]. Specifically, that study showed that the Sprint intervention based on training clinicians to use EHRs more efficiently, redesigning the multidisciplinary workflow within the clinic, and building new specialty-specific EHR tools both improved teamwork (ie, feelings of providing excellent care with the EHRs) and reduced clinician burdens.

The importance of the multidisciplinary approach for alleviating EHR-associated burdens has also been well documented by work conducted at the University of Toronto [25]. An EHR-SWAT Team representative of clinical and pharmacy informatics, health-information management, clinical applications, and project management, which was defined by the authors as a “low-cost alternative” to the Sprint initiative mentioned above, was set up to collect EHR challenges and physician requests and then prioritized and responded to them [25]. This “case report” identified supplementary EHR education (eg, finding documentation and information within the EHR and navigating notifications and confirmations within the EHR) as a main request. Divisional meetings according to clinical units were followed by interventions and their assessments. Physicians taking part in the program noted that it increased their proficiencies in using EHRs; indeed, most of them (79%) stated that they would recommend the program to colleagues.

Moreover, the engagement of all stakeholders is crucial to success. The same Canadian group conducted a cross-sectional survey using speech-recognition technology to assess the impact of the integrated Promoting Action on Research Implementation in Health Services (i-PARIHS) framework—which aims to guide the implementation of health care technologies—on EHR-related burdens in a mental-health context (at the Center for Addiction and Mental Health) [22]. The i-PARIHS framework includes four components related to innovation, facilitation, patient (ie, present or not) and context (eg, hospital, home, etc). Overall, two-thirds of the respondents were interested in using speech-recognition technology, but most mentioned relevant issues to be considered with respect to the four components of the framework such as the need for ongoing training, the fact that speech-recognition technology must recognize different accents, the possibility that speech-recognition technology could be uncomfortable for some patients, and the appropriateness of the environment regarding privacy and confidentiality. These insights emphasize the importance of personalizing digital tools according to the specific needs of HCPs and their patients. From these opinions and exploratory approaches, one may consider that EHR acceptability by HCPs implies understanding physicians’ desires and needs, engaging information technology and clinical teams in the decision-making process to make the implementation relevant, and periodically reviewing initiatives and synergistic opportunities within the strategy [20]. In the specialties concerned, engaging nurses in the organization and co-design of EHR-associated interventions is expected to both optimize EHR use and reduce burnout within nurse teams [47].

## Discussion and Perspectives

Several studies, reviews, and expert opinions published in the past few years have pointed out the burden of health digitization for HCPs [19-21,23,35-39,41,42]. The need to enhance HCP competencies regarding the use of HIT has been largely addressed, but the way of helping HCPs to integrate the corresponding technologies into their practice to alleviate mental load and make them more acceptable is just an emerging issue. Few but relevant studies have been recently published, which mostly focused on the use of EHRs [22-25,39,47]. In this context and to extend what has been done, we believe it is important to fill the gap by a holistic approach including the place of AI technologies and their impact on acceptability by HCPs to define how to enhance the HIT benefit:load balance.

The digitization of health care is expected to facilitate and accelerate connections between HCPs and between HCPs and patients. At the level of the medical community, this new era of clinical practice requires the harmonization and evidence-based validation of the digital tools [4,14,48-50]. At the individual level of each HCP, it implies adaptation in terms of knowledge, competence, time management, and relationships with patients and medical staff. Ongoing training and the development of a strong digital-therapeutic alliance between HCPs and patients will be crucial in bridging the gap between technology and clinical practice and ensuring that these solutions not only are integrated effectively but also enhance the overall quality of patient care [51]. Additionally, a major challenge is the development of a new digital semiology of symptoms that should be incorporated into medical-school courses and will allow equipping future health care professionals with the skills required to effectively assess and interpret digital health data. In the era of digitization of health care, the direct and indirect impacts on HCPs may jeopardize the acceptability of digital tools and AI due to new dimensions of responsibility, expanded time of work, lack of training, and organizational consequences.

The acceptability of digital tools in health care is a multifaceted concept that can be effectively analyzed using some tools such as the Technology Acceptance Model. This model developed by F.D. Davis [52] identifies two key dimensions: perceived usefulness and perceived ease of use. Perceived usefulness refers to whether the tool enhances HCP efficiency and performance. If the tool is seen as beneficial for improving patient care or reducing administrative burdens, it is more likely to be accepted. Conversely, perceived ease of use reflects how intuitive the tool is. A complex interface can increase cognitive load and thus lower its acceptability. In addition to the dimensions identified in the Technology Acceptance Model, several factors are expected to significantly influence the overall acceptability of digital tools in health care [53]. For one, efficiency is crucial because tools that streamline workflows and enhance productivity are more likely to be embraced by HCPs. Cognitive load is also critical. Tools that are overly complex can lead to frustration and decreased acceptance. Thus, designing with the user experience in mind can help reduce cognitive demands. Furthermore, time investment plays a vital role because HCPs are more inclined to adopt tools that require minimal training and provide immediate, tangible benefits. Moreover, cost considerations impact acceptability. If the financial burden of adopting a new technology exceeds its perceived benefits, HCPs may resist using it, regardless of its utility. Finally, trust in technology is a critical factor in digital-tool adoption. HCPs must trust the system’s reliability and ability to safeguard sensitive patient data. Failures in data security or system reliability can erode this trust and deter adoption. Trust is further influenced by the protection of personal health information, which is essential in a context of data breaches that could undermine the integrity of the HCP-patient relationship. Overall, these factors collectively shape the willingness of HCPs to integrate digital tools into their practice, and also support a composite tailored approach for technology acceptance.

Furthermore, ethical considerations are also paramount [40]. The tools must adhere to high standards of “data confidentiality” and “algorithm transparency.” HCPs must be confident that these technologies will not compromise patient privacy or perpetuate inequities in care access. To evaluate acceptability, several methods are recommended, including “standardized questionnaires” (eg, the Unified Theory of Acceptance and Use of Technology [UTAUT] model), “qualitative interviews,” and “usage data analysis” [54]. These provide valuable insights into both user perceptions and actual technology use. Finally, involving end-users in the design process ensures that the tools meet their practical needs and improves usability and fosters greater adoption. In that respect, the co-creation of digital tools with HCPs coupled with digital pedagogy can significantly enhance their acceptability and HCP well-being.

In addition to addressing the technical aspects, promoting digital well-being and offering support systems for HCPs should be prioritized to mitigate the negative effects. In that respect, implementing digital tools to make patient management more efficient requires a better understanding of such tools’ impacts on individual HCPs, ways to assess these impacts in practice, and the potential methods to optimize the benefit-risk ratio of health digitization for both patients and HCPs. Except for research on preventing or limiting the impact of EHRs, little has been documented regarding the optimization of the whole-health digital approach. In this context, projects developed to define the factors and dimensions that impact digital environments in health should be encouraged.

Based on the sate-of-the-art presented herein and to go further, the co-authors of this article have set up a task force including physicians specializing in digital health, mental health (eg, psychiatrists and a specialist in addictology), and chronic disease (eg, nephrologists and cardiologists), psychologists, researchers in the fields of digital health and cognition, a coordinator of digital health and AI within hospitals, and members of open-innovation platforms. Indeed, startups involved in the development of digital tools need to ensure that the solutions match the users’ needs. Such multidisciplinary approach is expected to provide a comprehensive understanding of the challenges posed by digital tools in health care. This so-called E-health Efficiency Evaluation (E3) project will involve four steps: (1) characterization of the impacts of digital tools, (2) identification of the critical factors/dimensions interfering with efficiency and acceptability of the digital solutions by HCPs and patients, (3) estimates of the extent of the different dimensions, and (4) assessment of the quality of a digital solution regarding these dimensions. The dimensions of interest will be defined according to subcategories including cognition, physical load, organizational load, and quality of life in taking into account the complex factors involved in EHR-related well-being among physicians [35]. An ongoing preliminary survey of HCPs has been initiated in order to better understand the relation between digital tools and quality of life at work,

The present overview focused on the impact of HIT on the mental and physical status of HCPs. Other related fields of investigation should be considered to optimize digital intervention and acceptability that include the mitigation of digital digitization-associated risks for malpractice and medical errors. The connection with patients in terms of experience and trust in HCP, especially regarding the role of AI in the decision-making process, is another major challenge.

## Conclusions

Over the past several years, it has become evident that alleviating and preventing negative impacts of digitization in health care must be considered according to a holistic approach including work-sharing, optimized workflows, individual training based on personal skills, and improvement of available tools, especially in terms of homogenization. Studies focused on interventions aimed at alleviating the burden induced by EHR have paved the way for multidisciplinary projects designed to contribute to a better understanding of the mental components impacted by digitization of care in the broad sense and to propose corresponding ways of mitigating those impacts in order to optimize both the overall benefits of digital tools in practice and their acceptability by HCPs. We are no longer at the beginning of the story, but there is still much to be done to fill the gap.

## Abbreviations

AI: artificial intelligence
EHR: electronic health record
HCP: health care professional
HIT: health-information technology
i-PARIHS: integrated Promoting Action on Research Implementation in Health Services
LLM: large language model
UTAUT: Unified Theory of Acceptance and Use of Technology
