# Correction: Acceptability of Health Information Technology by Health Care Professionals: Where We Are Now and How We Can Fill the Gap

**DOI:** 10.2196/89383

**Published:** 2026-01-06

**Authors:** Corinne Isnard-Bagnis, Stéphane Mouchabac, Riadh Lebib, Hervé Bismut, Pierre Alexis Geoffroy

**Affiliations:** 1 Nephrology Department, Pitié-Salpêtrière Hospital, AP-HP, Sorbonne Université, 47-83 boulevard de l'Hôpital, Paris, 75013, France, 33 1-4217-7211; 2 Academic Digital Medical Hub, AP-HP, Paris, France; 3 Department of Psychiatry, Saint-Antoine Hospital, AP-HP, Sorbonne Université, Paris, France; 4 Infrastructure for Clinical Research in Neurosciences (iCRIN), Paris Brain Institute, Paris, France; 5 Humans Matter, Paris, France; 6 Geminicis, Paris, France; 7 Département de psychiatrie et d'addictologie, AP-HP, GHU Paris Nord, DMU Neurosciences, Hôpital Bichat-Claude-Bernard, Paris, France; 8 Centre ChronoS, GHU Paris psychiatrie & neurosciences, Paris, France; 9 Université Paris Cité, Inserm, NeuroDiderot, Paris, France

In “Acceptability of Health Information Technology by Health Care Professionals: Where We Are Now and How We Can Fill the Gap” [1], the authors noted incomplete affiliations for authors CIB, SM, and PAG.

The previous list of affiliations was as follows:

Corinne Isnard Bagnis, MD, PhD^1^, Stéphane Mouchabac, MD^2^, Riadh Lebib, PhD^3^, Hervé Bismut, PhD^4^, Pierre A Geoffroy, MD, PhD^5^

^1^Department of Nephrology, Pitié-Salpêtrière Hospital, APHP Sorbonne Université, Paris, France

^2^Department of Psychiatry, Saint-Antoine Hospital, APHP Sorbonne Université, Paris, France

^3^Humans Matter, Paris, France

^4^Geminicis, Paris, France

^5^Department of Psychiatry, Hôpital Bichat-Claude-Bernard, Paris, France

This has been revised to:

Corinne Isnard Bagnis, MD, PhD^1,2^, Stéphane Mouchabac, MD^3,4^, Riadh Lebib, PhD^5^, Hervé Bismut, PhD^6^, Pierre A Geoffroy, MD, PhD^7-9^

^1^Nephrology Department, Pitié Salpêtrière Hospital, AP-HP, Sorbonne Université, Paris, France

^2^Academic Digital Medical Hub, AP-HP, Paris, France

^3^Department of Psychiatry, Saint-Antoine Hospital, AP-HP, Sorbonne Université, Paris, France.

^4^Infrastructure for Clinical Research in Neurosciences (iCRIN), Paris Brain Institute, Paris, France

^5^Humans Matter, Paris, France

^6^Geminicis, Paris, France

^7^Département de psychiatrie et d'addictologie, AP-HP, GHU Paris Nord, DMU Neurosciences, Hôpital Bichat - Claude-Bernard, Paris, France

^8^Centre ChronoS, GHU Paris psychiatrie & neurosciences, Paris, France

^9^Université Paris Cité, Inserm, NeuroDiderot, Paris, France

These corrections will appear in the online version of the paper on the JMIR Publications website, together with the publication of this correction notice. Because these were made after submission to PubMed, PubMed Central, and other full-text repositories, the corrected article has also been resubmitted to those repositories.

## References

[1] Isnard-Bagnis C, Mouchabac S, Lebib R, Bismut H, Geoffroy PA (2025). Acceptability of health information technology by health care professionals: where we are now and how we can fill the gap. J Med Internet Res.

